# Exploring the lung-gut direction of the gut-lung axis in patients with ARDS

**DOI:** 10.1186/s13054-024-04966-4

**Published:** 2024-05-27

**Authors:** Mairi Ziaka, Aristomenis Exadaktylos

**Affiliations:** 1 Clinic of Geriatric Medicine, Center of Geriatric Medicine and Rehabilitation, Kantonsspital Baselland, Bruderholz, Switzerland; 2 Department of Emergency Medicine, Inselspital, University Hospital, University of Bern, Bern, Switzerland

## Abstract

Acute respiratory distress syndrome (ARDS) represents a life-threatening inflammatory reaction marked by refractory hypoxaemia and pulmonary oedema. Despite advancements in treatment perspectives, ARDS still carries a high mortality rate, often due to systemic inflammatory responses leading to multiple organ dysfunction syndrome (MODS). Indeed, the deterioration and associated mortality in patients with acute lung injury (LI)/ARDS is believed to originate alongside respiratory failure mainly from the involvement of extrapulmonary organs, a consequence of the complex interaction between initial inflammatory cascades related to the primary event and ongoing mechanical ventilation-induced injury resulting in multiple organ failure (MOF) and potentially death. Even though recent research has increasingly highlighted the role of the gastrointestinal tract in this process, the pathophysiology of gut dysfunction in patients with ARDS remains mainly underexplored. This review aims to elucidate the complex interplay between lung and gut in patients with LI/ARDS. We will examine various factors, including systemic inflammation, epithelial barrier dysfunction, the effects of mechanical ventilation (MV), hypercapnia, and gut dysbiosis. Understanding these factors and their interaction may provide valuable insights into the pathophysiology of ARDS and potential therapeutic strategies to improve patient outcomes.

## Introduction

ARDS represents a severe form of respiratory failure characterized by acute hypoxaemia and bilateral radiographic infiltrates due to lung inflammation and excessive alveolocapillary permeability, not attributed to cardiogenic pulmonary edema [1]. In 1821, Laennec documented cases of fatal "idiopathic pulmonary edema," marking the earliest known recording of the syndrome. During the First and Second World Wars, cases were observed in which different traumatic injuries could result in lung oedema over time. This led to the introduction of the term "shock lung" to describe the phenomenon. In 1967, Ashbaugh and colleagues published a case series involving 12 patients who developed respiratory failure in the context of various clinical conditions, representing the first systematic presentation of the syndrome [2, 3]. Since then, the clinical definition of ARDS has undergone significant revisions. Initially, an American–European consensus conference was convened in 1992 by the American Thoracic Society and the European Society of Intensive Care Medicine [4], followed by further revisions led by the ARDS Definition Task Force in Berlin in 2012 under the guidance of the European Society of Intensive Care Medicine [5, 6]. These revisions aimed to optimize the focus on the syndrome’s radiological manifestations and the severity of oxygenation impairment [3, 6]. The current definition, known as the Berlin definition, specifies that at the time of diagnosis, patients must receive at least 5 cmH_2_O of positive end-expiratory pressure (PEEP) [5, 6].

ARDS is a common condition, with epidemiological studies indicating that over three million individuals worldwide are diagnosed with it annually, constituting approximately 10% of those admitted to intensive care units (ICUs) [7]. Despite significant recent progress in treatment modalities and supportive care for ARDS patients, which includes the adoption of extracorporeal membrane oxygenation and protective lung ventilation strategies [8], the mortality rate associated with ARDS remains high, ranging from 34.9 to 46.1% [9]. In critically ill patients, hypoxia and/or hypercapnia seldom directly contribute to mortality. Today it is well established, that rather it emerges from the initiation of a systemic inflammatory response, leading to MODS and its associated complications. Notably, significant attention has been directed toward the role of the gastrointestinal (GI) tract in the pathogenesis of this syndrome [10]. Indeed, the significant challenge for clinicians and researchers is associated with the diverse nature of ARDS, which is evident in its various causes, manifestations, and responses to therapy. This complexity underscores the necessity for precise supportive care and the exploration of potential therapeutic strategies [11, 12].

The gut is recognized to have a substantial impact on critical illness, including trauma, pancreatitis, haemorrhagic shock, burns, and ARDS, by modifying systemic inflammation and pathogenesis of sepsis with a pivotal role in the pathophysiology of MODS in critically ill patients [13–17]. Previous theories suggested that intestinal hyperpermeability results in bacterial translocation into the systemic circulation in critical illness. However, today, it is widely recognized that the pathophysiological mechanisms involved are more complex than previously assumed. Indeed, critical illness impacts all aspects of the gut, including the epithelium, the immune system, and the microbiome, thus potentially initiating a pathological host response reaction [18]. Investigation of organ crosstalk in critical illness has revealed multifactorial biological communication between different organ systems, including gut-lung crosstalk [19]. Certainly, mounting evidence demonstrates the role of the gut microbiome in critical illness, including its impact on the onset, progression, and outcomes of the underlying disease [20]. It has been suggested that gut bacteria may enter the lungs through translocation during critical illness, facilitated by increased gut and alveolo-capillary permeability. This process can influence disease progression, drug response, and systemic inflammatory reactions and contribute to organ dysfunction. Among mechanically ventilated patients, there is an observed enrichment of lung microbiota with gut-associated microbes, which has been linked to the development of ARDS [21, 22]. However, the impact of LI/ARDS on gut homeostasis and function in patients with LI/ARDS has received limited attention. This review investigates recent advancements in understanding the interplay between the lungs and the gut in patients with LI/ARDS. Emphasis is placed on systemic and local inflammation, epithelial barrier dysfunction, effects of MV, hypercapnia, and gut microbiome dysbiosis, all of which are believed to influence lung-gut interactions significantly. In light of our recent comprehensive research into the gut-lung axis, as presented in our work on the triple hit hypothesis of LI in patients with acute brain injury [23], this review exclusively focuses on elucidating the lung-gut direction of the bidirectional communication between the lungs and the gut in LI/ARDS.

## Impact of systemic inflammation on gut integrity in patients with ARDS

**Fig. 1:**
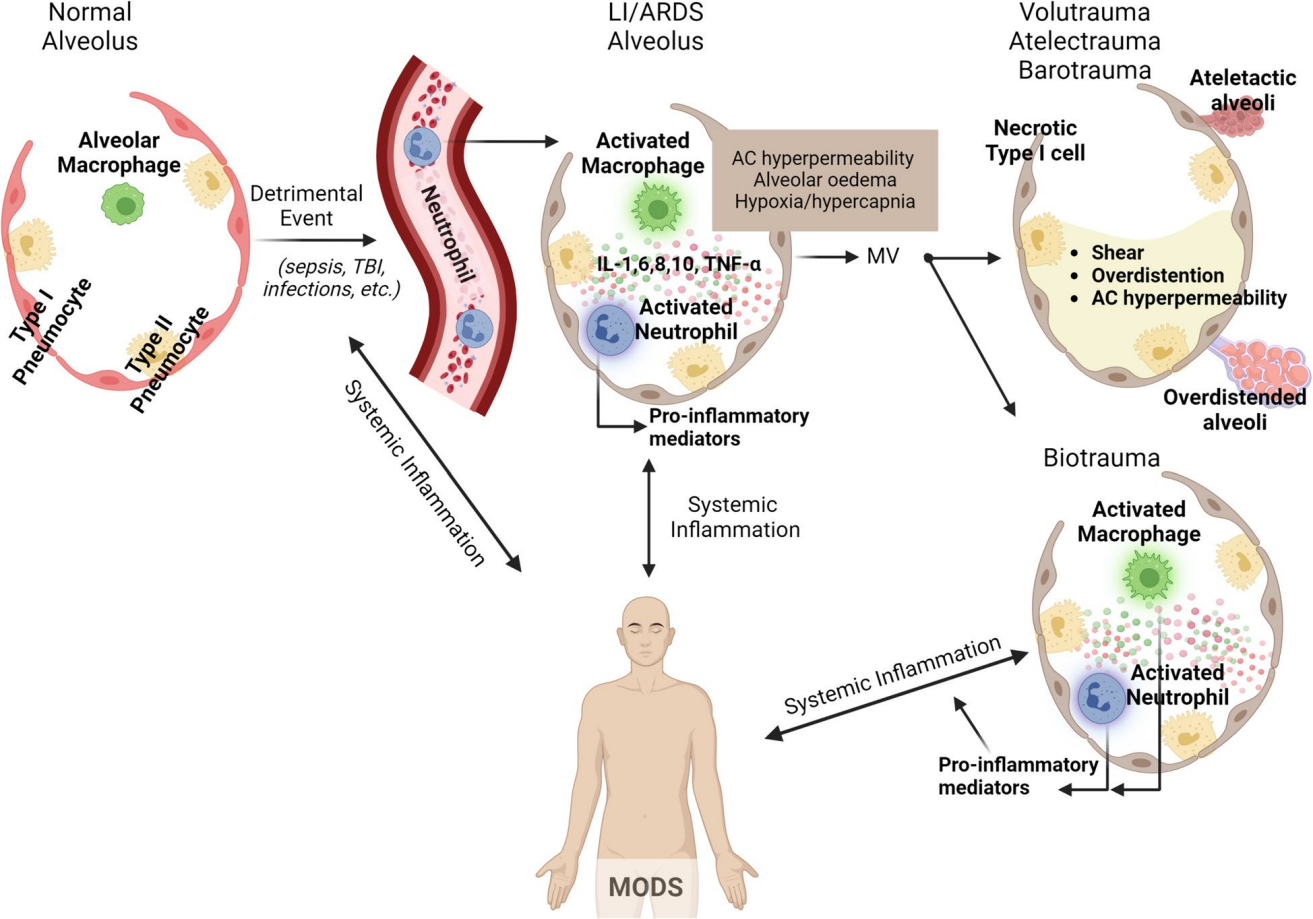
In patients with LI/ARDS, damage to the endothelium of pulmonary capillaries occurs, leading to the migration of activated immune cells into the lungs, thereby exacerbating the pulmonary inflammatory response. Within the air space, alveolar macrophages locally secrete cytokines to induce chemotaxis and activate neutrophils, which, in turn, release various pro-inflammatory molecules. The use of MV may further worsen lung injury, potentially resulting in excessive alveolar distension (volutrauma), the repetitive cyclic opening and closure of alveoli (atelectrauma), and the initiation of a complex inflammatory cascade, leading to both local and systemic inflammation (biotrauma). This inflammation can extend to distant organs and systems, exacerbating multiple organ dysfunction. *ARDS* acute respiratory distress syndrome, *IL* interleukin,* LI* lung injury, *MODS* multiple organ dysfunction syndrome, *MV* mechanical ventilation, *TBI* traumatic brain injury, *TNF* tumor necrosis factor

Severe acute inflammation plays a fundamental role in the pathogenesis of LI/ARDS [24]. Dysregulated systemic inflammation, marked by a sustained increase in circulating inflammatory cytokines and chemokines, is recognized as the central pathogenetic process leading to organ dysfunction and failure in ARDS patients (Fig. 1) [25]. Indeed, ARDS is characterized by two-way interactions between the lungs and other organ systems, rather than being localized solely to the pulmonary system, manifesting as a systemic inflammatory condition associated with elevated levels of inflammatory cytokines. Certainly, interleukin (IL)-1β, tumor necrosis factor (TNF)-α, IL-6, and IL-8 are observed in both bronchoalveolar lavage fluid (BALF) and circulating plasma of individuals with ARDS [25]. This abnormal inflammatory response is a primary driver of both short-term and long-term morbidity and mortality in this patient population [25, 26], prompting tissue alterations in vital organs, culminating in the development of MODS (Fig. 1) [27]. Longitudinal evaluations of inflammatory cytokine levels have revealed that systemic and pulmonary inflammation persists for several weeks and may, in severe cases, extend for long periods of time after the clinical resolution of respiratory failure and extubation [25]. However, recent data regarding the duration of pro-inflammatory responses, particularly in the context of increased steroid use for septic shock, severe community-acquired pneumonia (CAP), and ARDS as per current recommendations and studies [28], remains unclear.

It has been suggested that specific etiologies of ARDS are associated with an elevated incidence of systemic inflammation. Particularly, sepsis, traumatic brain injury (TBI), and burn injury are identified as primary instigators of LI/ARDS within the ICU, with severe acute inflammation playing a pivotal role in its pathogenesis [29–33].

**Fig. 2:**
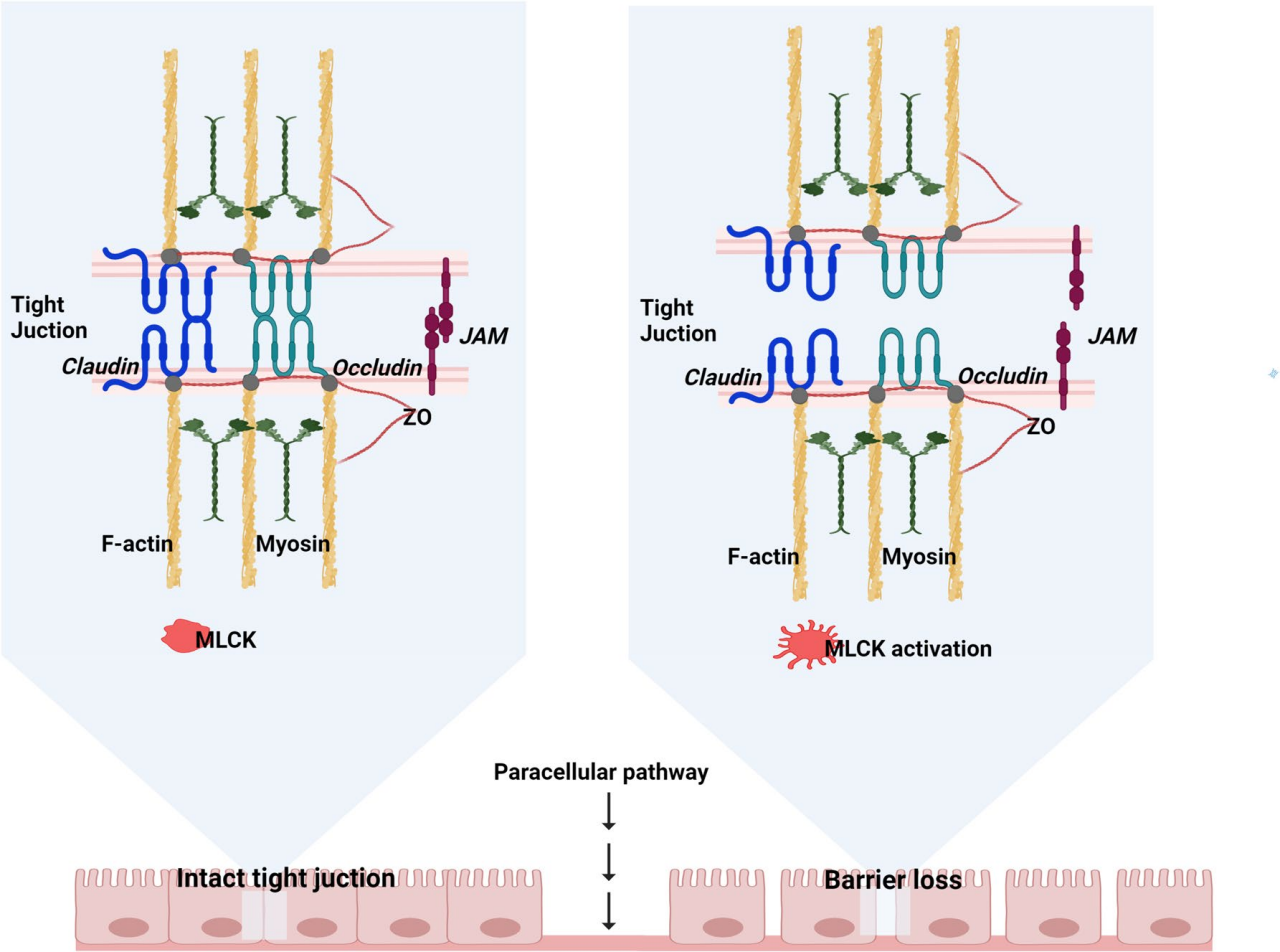
The role of the gut in both health and critical illness. In critical illness, alterations in TJ integrity, which is vital for maintaining homeostasis, lead to loss of gut integrity and increased permeability. *JAM* junctional adhesion molecules, *MLCK* myosin light chain kinase, *ZO* zonula occludens

In the context of sepsis-associated LI/ARDS, enhanced production and augmentation of inflammatory cytokines have been associated with intestinal barrier dysfunction [34] and a significant increase in intestinal permeability, closely linked to the onset and progression of sepsis [35], and disrupted mucous layer integrity, characterized by compromised adhesion, reduced thickness, and decreased lumen coverage [36]. Indeed, cytokine storm leads to intestinal hyperpermeability through functional alterations of claudins, particularly up-regulation of junctional adhesion molecules (JAM) and claudin 2 and down-regulation of claudin 5 [37] and distribution of claudins 1, 3, 4, 5, and 8 as well, resulting in intestinal barrier dysfunction [38], disrupted paracellular transport of solutes and increased permeability to macromolecules (Fig. 2) [39]. Moreover, paracellular hyperpermeability is further mediated by cytokine-associated activation of myosin light chain kinase (MLCK), with cytokines further amplifying MLCK activity through a feed-forward mechanism, partly via alterations in claudin 15 [40] (Fig. 2). Similarly, in prospective cohort studies, patients with TBI displayed severe hypercytokinaemia (IL-1β, IL-6, IL-8, IL-10, and TNF-α) [33]. Elevated levels of TNF-α, IL-1β, and IL-6 in patients with TBI can impact the permeability of tight junctions (TJ) in the intestine [41]. Indeed, TNF-α binds to TNF receptors (TNFRs) on intestinal epithelial cells, initiating the upregulation of molecular pathways associated with pro-inflammatory cytokines like nuclear factor kB (NF-kB). Additionally, sympathetic hyperactivation following acute brain injury induces splanchnic hypoperfusion, leading to modifications in intestinal TJ proteins such as occludin and zonula occludens-1 (ZO-1), ultimately corresponding to intestinal hyperpermeability (Fig. 2) [42, 43].

Recent research supports the hypothesis that impaired intestinal permeability leads to the bacterial translocation of gut microbes, such as *Bacteroidetes* and *Enterobacteriaceae*, entering the lungs of ARDS patients [44]. The translocation of bacteria initiates local activation of the mucosal immune system (MIS), prompting the production of inflammatory compounds (danger-associated molecular patterns-DAMPs) that traverse the mesenteric lymphatics to enter the lung and systemic circulation. Recognition of these molecules by innate immune cells fosters additional pro-inflammatory pathways, accelerating the progression of organ damage and MODS, including the gut [35, 45, 46].

## Impact of mechanical ventilation on gut integrity

**Fig. 3:**
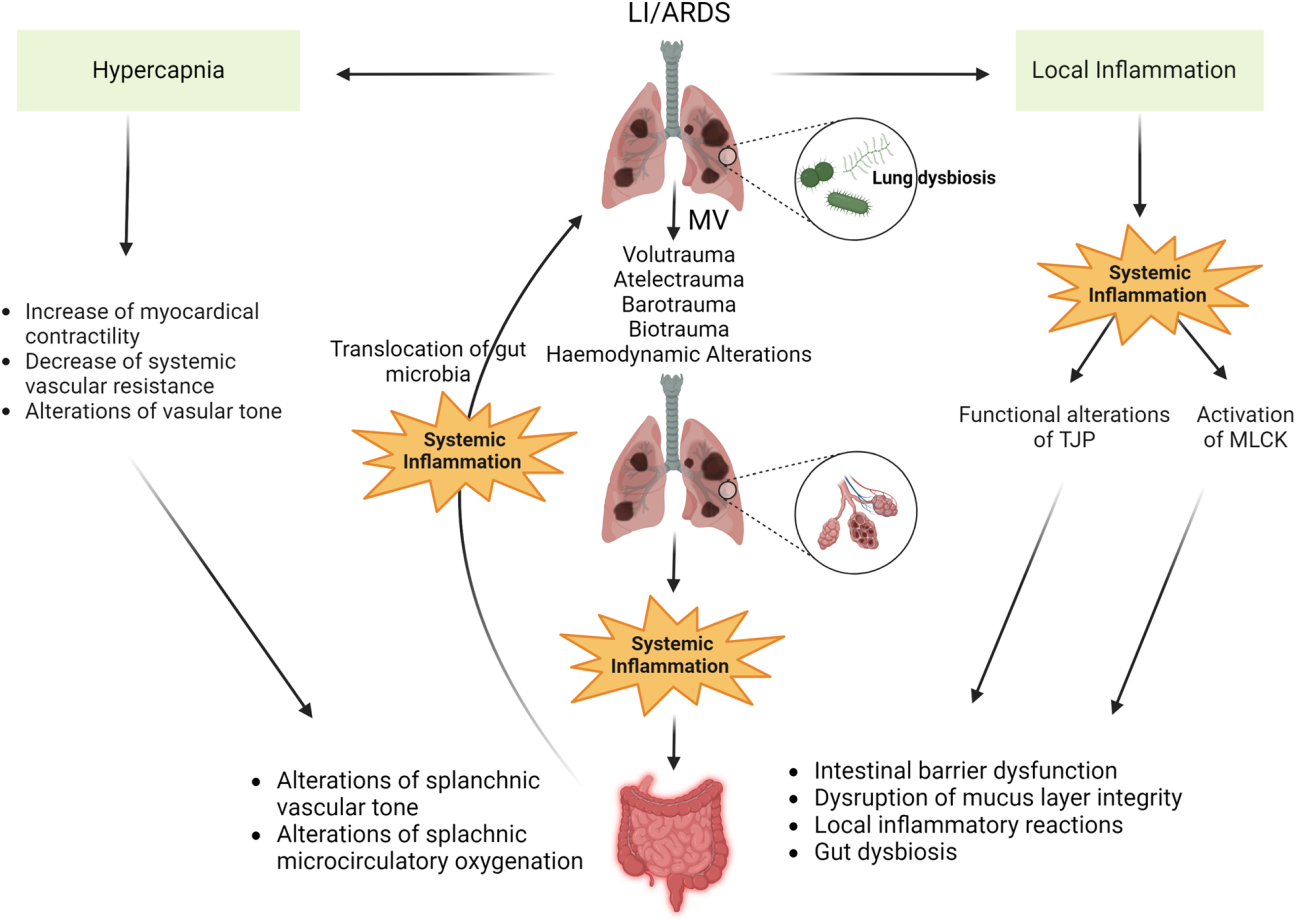
In patients with LI/ARDS, severe acute inflammation leads to functional alterations of tight junction proteins and activation of MLCK, which are associated with intestinal barrier dysfunction, disruption of the mucus layer integrity, exacerbation of intestinal inflammatory reactions, and gut dysbiosis. MV may cause VILI, further exacerbating systemic inflammation and intestinal damage. Haemodynamic alterations during low-tidal volume MV and hypercapnia induced by protective MV are associated with increased myocardial contractility, decreased systemic vascular resistance, and alterations of vascular tone, leading to changes in splanchnic vascular tone and splanchnic microcirculatory oxygenation. Intestinal dysfunction and loss of gut integrity enhance systemic inflammation and promote the translocation of gut bacteria into the lungs, worsening pre-existing LI-mediated lung dysbiosis. *ARDS* acute respiratory distress syndrome, *LI* lung injury, *MLCK* myosin light chain kinase, *MV* mechanical ventilation, *TJP* tight junction proteins, *VILI* ventilator-induced lung injury

Although MV is considered one of the cornerstones of ARDS management, the recognition that it can contribute to LI represents a significant advancement in ARDS research [23, 24, 47]. Termed ventilator-induced lung injury (VILI), this phenomenon involves a range of mechanisms, including exposure to elevated inflation transpulmonary pressures (barotrauma), excessive alveolar distension (volutrauma), and repetitive cyclic opening and closure of alveoli (atelectrauma). Furthermore, beyond causing direct structural alterations, these mechanical forces can trigger a complex inflammatory cascade, resulting in both local and systemic inflammation (biotrauma) [48], potentially extending to distant organs and systems, exacerbating multiple system organ dysfunction and, ultimately, contributing to elevated mortality (Figs. 1, 3).

The pathophysiologic participation of biotrauma in the pathogenesis of VILI remained unclear until 1998 [48]. In recent years, it has become increasingly evident that under specific conditions, MV can provoke inflammatory responses and subsequent lung injury while effectively maintaining gas exchange. These inflammatory cascades have the potential to disseminate throughout the systemic circulation, exerting effects on extrapulmonary organs and systems, ultimately culminating in MOF [49]. Indeed, it is assumed that by increasing alveolar–vascular permeability, MV facilitates the decompartmentalization of the inflammatory response and is instrumental in the propagation of the injury from the lung to the distal organs, ultimately resulting in MOF and impacting mortality (Figs. 1, 3) [49]. Moreover, it is hypothesized that the MV-induced inflammatory response (biotrauma) arises from two distinct pathophysiologic mechanisms. Firstly, direct cellular trauma disrupts cell walls, leading to the release of cytokines both locally at the alveolar level and into the systemic circulation [50]. Secondly, a mechanism termed “mechanotransduction” has been identified, referring to the phenomenon that cells detect mechanical forces through a process known as "mechanosensation" and convert them into a series of cellular signaling events through "mechanotransduction," In vitro studies have highlighted that the majority of pulmonary cells can produce cytokines in response to cyclic stretch [51]. Furthermore, mechanical injury results in the release of DAMPs, which in turn trigger the recruitment of immune cells that produce pro-inflammatory cytokines. This, coupled with the activation of signaling cascades in alveolar epithelial and vascular endothelial cells due to overstretching, as well as dysregulation of the neuroinflammatory reflex, leads to a potent systemic inflammatory response. The continuous exposure to harmful mechanical forces during MV, especially high tidal volume (TV) MV, leads to a vicious cycle by exacerbating this inflammatory process, leading to increased permeability of the alveolar-capillary barrier, and enhancing susceptibility to additional lung damage [52].

Clinical studies have shown associations between MV and increased levels of inflammatory mediators such as TNF-α, IL-1, IL-6, IL-8, soluble TNF-α receptor 75, IL-1β, and IL-1 receptor antagonist (IL-1ra) in both plasma and BALF [47, 53, 54]. These findings are further supported by experimental studies demonstrating that MV may trigger the release of various proinflammatory mediators including IL-1β, IL-6, IL-8, TNF-α, C-X-C motif ligand 1 (CXCL1), and CXCL10, as well as macrophage inflammatory protein-2 [55], and intercellular adhesion molecule levels [56–59].

Several studies have attempted to elucidate the pathophysiology of distal organ injury in ARDS; however, the exact mechanisms remain incompletely understood. These potential factors include MV, hypoxaemia, the deleterious effects of bacteria or bacterial products, and systemic inflammation [60, 61]. Indeed, as mentioned above, accumulating evidence supports the hypothesis that ventilator-mediated inflammatory reactions and injury may extend to distal organs such as the liver, kidney, and brain [62, 63]. Nevertheless, little is known about the injurious effects of VILI on gut integrity. Moreover, the existing evidence regarding gut damage and dysfunction as possible detrimental consequences of MV-induced systemic inflammation is limited. As a component of the inflammatory/sepsis cascade, increased levels of TNF-α, IL-1β, and IL-6 have been shown to impact the permeability of TJ in the intestine [41]. Guery and coworkers (2003) have shown that MV elevates plasma TNF levels and gut permeability and that the administration of a TNF-neutralizing antibody intravenously reversed gut hyperpermeability [64]. The detrimental effects of TNF-α on the integrity of the intestinal epithelial barrier—independently or in conjunction with other proinflammatory cytokines—have been indicated by numerous prior investigations focusing on both experimental models and cell culture settings [65–68]. The underlying mechanisms of TNF-α-induced dysfunction of the intestinal epithelial barrier are complex and enclose variant processes: the induction of apoptosis in intestinal epithelial cells, alterations in the lipid composition of cell membranes, activation of MLCK by calcium-calmodulin, stimulation of myosin light chain (MLC) phosphorylation through increased expression of MLCK protein, and suppression of TJ protein expression. Notably, as pivotal in developing TNF-α-induced dysfunction in the intestinal epithelial barrier, the MLCK-mediated MLC phosphorylation pathway has been widely recognized [69]. Moreover, IL-6 is a multifunctional pro-inflammatory cytokine with pleiotropic effects, participating significantly in inflammatory responses both locally in the gut and systemically. However, despite its crucial role in inflammatory processes, the exact impact of IL-6 on the modulation of intestinal epithelial barrier function remains uncertain, as there is controversy regarding whether IL-6 exerts a protective or disruptive effect on the intestinal barrier [41]. Very recently, using an experimental mice model of VILI, Ding et al. [70] demonstrated elevated levels of TNF-α, IL-1β, and IL-6 in both serum and gut tissues, as assessed by enzyme-linked immunosorbent assay (ELISA). Remarkably, VILI mice exhibited significant increases in gut injury and a phenomenon known as PANoptosis, referring to the simultaneous occurrence of apoptosis, pyroptosis, and necroptosis in the gut, which is associated with MLC activation and TJ disruption. These pathological changes were associated with elevated TNF-α, IL-1β, and IL-6 serum levels. Moreover, VILI mice present impairments in intestinal barrier integrity, characterized by reduced expression of occludin and ZO-1, along with increased expression of claudin-2 and activation of MLC. Notably, intratracheal administration of Importin-7 (Imp7) small interfering RNA (siRNA) nanoparticles effectively suppressed the production of cytokines. It mitigated gut injury induced by VILI, suggesting the pathophysiologic effects of systemic inflammation in exacerbating gut injury following VILI and indicating the potential therapeutic utility of Imp7 siRNA nanoparticles for cytokine inhibition as a treatment strategy for VILI-associated complications [70]. In addition, experimental studies using a rabbit model of VILI illustrate a potential mechanism for the influence of biotrauma on MOF. By utilizing an acid aspiration model of ARDS, Imai and co-workers (2003) revealed that harmful ventilatory strategies, such as high TV and zero PEEP, markedly elevated the apoptosis of epithelial cells in the kidneys and small intestine, elevation which correlated significantly with organ dysfunction [71].

## Impact of mechanical ventilation from a mechanistic point of view

The progressive deterioration of other organ systems, ultimately leading to the onset of MODS, is the primary factor contributing to elevated mortality among patients experiencing LI/ARDS [72]. Indeed, in critically ill patients, mortality is less frequently associated with hypoxia and/or hypercapnia but rather arises from the development of a systemic inflammatory response leading to MODS with related complications and even death. Notably, significant attention has been directed toward the role of the GI tract in the pathogenesis of this syndrome [10].

Numerous studies indicate that lung protective techniques involving low TV and elevated PEEP levels lead to decreased mortality rates, thereby becoming the standard management approach for patients with ARDS [24, 73], yet, existing data regarding the impact of PEEP on splanchnic perfusion and systemic haemodynamics are contradictory. Indeed, despite enhancing arterial oxygenation, PEEP's application may adversely affect systemic haemodynamics by decreasing venous return and cardiac output, with the extent of these effects correlating with the level of PEEP administered [74]. Indeed, experimental and clinical investigations have revealed that among mechanically ventilated individuals without lung injury, PEEP induces a decrease in venous return, consequently impacting cardiac output [75–77]. Moreover, it has been demonstrated that during abdominal surgery, increasing PEEP from 0 to 15 cm H_2_O is associated with a simultaneous reduction in mixed-venous and hepatic venous oxygen saturations, with significant changes observed only at a PEEP of 15 cm H_2_O [78]. Additionally, the application of PEEP in patients who underwent abdominal surgery, along with the decrease in cardiac output, also impairs portal blood flow. At the same time, splanchnic oxygen consumption remained maintained due to compensatory increases in splanchnic oxygen extraction [79]. Similarly, in patients with LI resulting from septic shock, elevating PEEP levels to 15 cm H_2_O led to reductions in both cardiac output and hepatic vein oxygen saturation [80], which was significantly prominent at a PEEP level of 15 cm H_2_O compared to 10 cm H_2_O [81]. Moreover, PEEP can autonomously affect regional perfusion, particularly hindering splanchnic perfusion. Impaired splanchnic perfusion may compromise the gut`s barrier function, resulting in intestinal hyperpermeability and bacterial translocation and facilitating the initiation of MOF [74]. Experimental studies have shown a dose-dependent impact of PEEP on splanchnic blood flow. At PEEP levels below 10 cm H_2_O, the reduction in splanchnic blood flow is usually limited, and it becomes more prominent at levels ranging from 15 to 20 cm H_2_O [82]. Conversely, a number of clinical studies in patients with ARDS have demonstrated that high PEEP levels, up to 20 cmH_2_O, do not compromise gastric mucosal perfusion, as evaluated by tonometry, and are not related to significant impairments of systemic haemodynamics in the majority of ARDS patients [74, 81, 83]. However, it should be emphasized that the impact of PEEP is intricate and difficult to predict, particularly in the context of heterogeneous ARDS lungs. The effects of PEEP are influenced not only by the selected PEEP level but also by how it interacts with and alters lung status [84]. Therefore, managing patients at the bedside requires achieving a careful equilibrium between lung recruitment and preventing hyperinflation, with close monitoring of the haemodynamic response [85].

While this section primarily focuses on the mechanistic influence of MV on splanchnic perfusion and systemic haemodynamic alterations affecting gut integrity, the effects of MV on gut function are multifactorial. Elevated plasma-renin–angiotensin–aldosterone activity and enhanced catecholamine levels due to sympathetic activation are among the additional mechanisms that notably contribute to splanchnic hypoperfusion [86]. Moreover, commonly used medications to support MV, like opiates and sedatives—particularly benzodiazepines—can decrease gastrointestinal motility and hinder venous return through mechanisms such as venodilation or decreased responsiveness to vasopressor agents [86].

Considering the data provided above and the necessity of lung protective strategies, such as employing low TV and high levels of PEEP, in patients with ARDS, it is crucial to closely monitor both systemic and regional perfusion when administering high PEEP levels. Furthermore, future investigations should focus on assessing the prolonged effects of PEEP on splanchnic perfusion. Understanding the complexity of interactions between critical illness and the mechanistic impact of MV on the GI tract enables appropriate management and prospective use of preventive practices.

## The role of hypercapnia within the lung-gut axis

Acute respiratory failure not only interrupts the process of gas exchange but also results in significant stress on cardiovascular function, necessitating higher cardiac output to maintain sufficient oxygen delivery. This challenge is exacerbated by enhanced oxygen demand resulting from increased breathing work and inefficient gas exchange. Additionally, lung collapse due to hypoxic pulmonary vasoconstriction contributes to increased right ventricular afterload, further complicating cardiovascular function [87]. Lung protective ventilation, characterized by using a small TV, is frequently associated with hypercapnia in patients with LI/ARDS [24, 26]. In the context of acute respiratory acidosis, pleiotropic cardiovascular consequences may trigger increased myocardial contractility (Fig. 3). This may be done through sympathetic nerve activation and simultaneous reduction in systemic vascular resistance. Moreover, changes in local vascular tone leading to vasoconstriction in the lungs and kidneys, alongside vasodilation in the brain show that regional perfusion may undergo dynamic alterations [10, 88]. In a biphasic manner, hepatic and splanchnic blood flow seems to be influenced by hypercapnia. In the beginning, sympathetic stimulation leads to a reduction in blood flow, followed by a subsequent increase attributed to the direct vasodilatory effect of carbon dioxide (CO_2_). Furthermore, it has been found that moderate fluctuations in CO_2_ arterial partial pressure (PCO_2_) correlate with elevated systemic perfusion in mechanically ventilated patients under stable conditions. Splanchnic perfusion, however—assessed by the difference in gastric mucosal/arterial PCO_2_ (DPCO_2_)—remained unaltered [10]. Experimental studies support these findings further by showing that in septic animal models, splanchnic microcirculatory oxygenation is enhanced by both acute hypercapnic acidosis and buffered hypercapnia, effectively mitigating the negative impacts induced by sepsis [89, 90]. On the contrary, further clinical studies have shown that lung-protective ventilation strategies in patients with ARDS failed to enhance gastric mucosal perfusion despite leading to increased cardiac output. It is hypothesized that the variability noted in the individual alterations of gastric mucosal perfusion resulting from low TV MV suggests that the direct local vasodilation caused by elevated tissue PCO_2_ may be counteracted by the augmented release of catecholamines in the systemic circulation [91].

## Impact of ischaemia/reperfusion injury on gut integrity

Proposed in 2002 by Deitch, the "three-hit model" theory suggests a sequence of events in which an initial injury induces visceral hypoperfusion (first hit), prompting the gut to generate and release proinflammatory mediators. Subsequent haemodynamic resuscitation results in reperfusion, causing ischemia–reperfusion injury to the gut (second hit). This leads to loss of gut barrier function and an enhanced inflammatory reaction of intestinal origin, independent of bacterial or toxin translocation. Upon crossing the mucosal barrier, bacteria, and endotoxin further stimulate the immune response by releasing chemokines, cytokines, and other inflammatory mediators (Fig. 3). These substances exert their effects on the immune system, both locally and systemically, ultimately precipitating systemic inflammatory response syndrome (SIRS) and MODS, referred to as the third hit [13, 92]. Finally, as already mentioned, in the context of critical illness, damage occurs to the mucus layer, resulting in epithelial cell dysfunction. Ischaemia/reperfusion events further exacerbate this issue by diminishing the hydrophobicity of the mucus layer and causing changes in intestinal permeability [93].

## Impact of altered lung microbiome on gut integrity

The widespread notion of lung sterility posed a barrier to the systematic investigation of the lung microbiome, resulting in a slowdown of research progress [94, 95]. However, research has revealed the prevalence of microaspiration or gastroesophageal reflux, even among apparently healthy individuals, leading to microbial colonization of the alveoli [96–99]. Bronchoscopic studies have highlighted that the carina represents the densest site of bacterial deoxyribonucleic acid (DNA) among healthy individuals, with a gradually diminishing density with further bifurcations, suggesting the impact of micro-aspiration on microbial immigration in the respiratory tract of healthy adults [100]. Using culture-independent molecular techniques, a diverse bacterial community has been revealed in the lower airways of asymptomatic individuals, predominantly characterized by species such as *Prevotella, Veillonella, Streptococcus, and Fussobacterium* [101–103].

Critically ill patients could necessitate a range of interventions in the ICU, such as MV, antibiotic therapy, continuous blood purification, and immunosuppressive regimens [100], which potentially may influence the microbial composition and diversity of these patients [104–106]. Alterations of the lung microbiome associated with critical illness significantly correlate with systemic and local inflammation [44, 101, 104, 107]. A reduction in bacterial diversity occurs, and potential pathogens, often originating from alternative ecosystems like the GI tract and the skin, may displace commensal microbial populations [101, 108]. Examining BALF from patients with ARDS, Kyo et al. [109] discovered tendencies toward increased lung bacterial burden, evidenced by elevated 16S rRNA gene copy numbers. Furthermore, they observed a notable reduction in alpha diversity among ARDS patients, encompassing both copy numbers and the relative abundance of betaproteobacteria [109]. Moreover, patients with LI exhibit augmented contamination of gut-associated bacteria within their lung microbiome, including species from the *Enterobacteriaceae* family [44], which have been associated with the progression to ARDS [44]. Although the enrichment of the lung microbiome with gut-derived bacteria may suggest a broader dysbiosis in critically ill patients, a study conducted by Panzer et al. (2018) highlights the significant role of these microbes in ARDS pathophysiology [44]. The authors emphasize a substantial involvement of these bacteria in ARDS pathogenesis, particularly in patients who underwent MV, where early lung dysbiosis coincides with a notable increase in inflammatory mediators (IL-6, IL-8), thereby predisposing patients to subsequent ARDS development [44].

The composition of gut microbiota could be influenced by lung dysbiosis (Fig. 3). In a pre-clinical study, influenza infection was found to cause a rise in the presence of *Enterobacteriaceae* and a decline in the levels of *Lactobacilli* and *Lactococci* in the gut [110]. Moreover, a recent study by Gu and colleagues (2020) demonstrated that Coronavirus disease (COVID)-19 patients exhibited substantially decreased intestinal bacterial diversity compared to healthy controls. Additionally, there was a notable increase in the relative abundance of opportunistic pathogens like *Streptococcus*, *Rothia*, *Veillonella*, and *Actinomyces*, alongside a decrease in the relative excess of beneficial symbionts [111].

The intestinal microbiome undergoes significant alterations due to both the physiological impacts of critical illness, including sepsis and ARDS, and the clinical interventions employed in intensive care settings [104]. In a clinical study including 52 participants, rectal swabs were collected to assess the composition of the gut microbiome using 16S rRNA gene sequencing over their ICU stay. Additional research, including patients’ mortality rate at 28 days after admission to the ICU, revealed dysbiosis in the gut of critically ill patients. The observed dysbiosis was identified as an independent risk factor for increased mortality at 28 days, and, according to the authors, lower mortality was associated with genera (e.g., *Parasutterella* and *Campylobacter*), while high mortality with taxa from the *Anaerococcus* genus and *Enterobacteriaceae* family [112].

The profound impact of changes in the gut microbiome as a risk factor for LI/ARDS was acknowledged as early as the late 1970s. Cuevas and colleagues demonstrated in animal models of shock that the onset of lung injury could be avoided through pretreatment with enteric antibiotics [113]. Furthermore, Wang and colleagues (2014) demonstrated that in experimental influenza infection, the interferon (IFN)-γ produced by lung-derived CCR9 + CD4 + T cells altered the composition of the gut microbiota and induced intestinal immune injury [114]. Additional experimental studies further support the hypothesis that influenza pulmonary infection can substantially modify intestinal microbiota profile depending on a mechanism involving type I interferons (IFN-Is) [115]. However, data are scarce concerning the pathophysiology of how lung dysbiosis may influence the gut microbiome in patients with LI/ARDS. Given that the compromised permeability of the alveoli-capillary membrane increases in LI/ARDS due to direct (mainly epithelial) or indirect (mainly endothelial) damage, it is reasonable to speculate that alveoli-capillary permeability represents a significant risk factor for gut–lung bacterial translocation (Figs. 1, 3) [116]. Moreover, during critical illness, there is a significant restructuring of the environmental conditions that support gut bacterial growth, leading to a profound impact on the reproductive rates of microbial community members [104].

In host defense, mucus plays a fundamental role by forming a barrier that protects the gut epithelium from direct contact with bacteria and digestive enzymes. Due to its hydrophobic properties, mucus substantially hinders the passage of positively charged, water-soluble toxic molecules across its surface [93]. In addition, the proliferation of microbes within the *Proteobacteria* phylum is facilitated by elevated nitrate levels and a modified mucosal oxygen gradient. Included in this phylum are several widely recognized gram-negative rods such as *Pseudomonas aeruginosa* and *Escherichia coli*, along with specific members of the *Firmicutes* phylum like *Staphylococcus aureus* and *Enterococcus spp* [117–121]. Resulting from the transition to a “disease-promoting microbiome” or “pathobiome” in the context of critical illness, subsequent pro-inflammatory processes occur within the intestinal epithelial cells. This can present as enhanced permeability of TJ and breakdown of mucus integrity, both factors implicated in gastrointestinal injury and the development of MODS [122–124].

Ultimately, while mounting evidence suggests that changes in the lung microbiome could impact gut integrity, there remains a significant gap in our understanding of how lung dysbiosis specifically influences the gut microbiome in ARDS. Further investigation is crucial to elucidate these pathways and their clinical relevance.

## Therapeutic perspectives

Given the significant impact of the gut-lung axis and intestinal integrity on critical illness, interventions aimed at preserving and restoring the microbiota hold promise for enhancing outcomes in critically ill patients with ARDS. Bacterial metabolites from the intestine, particularly short-chain fatty acids (SCFAs), play a crucial role in influencing both local and systemic immune responses, thereby contributing significantly to the immunomodulatory effects of probiotics and prebiotics [125, 126]. The term “prebiotics” refers to dietary components, predominantly indigestible oligosaccharides, that specifically promote the growth and activity of beneficial gut microbiota, enhancing the balance of intestinal flora and contributing to improvements in human health [127]. Prebiotics include various dietary nutrients, such as carbohydrate-based dietary fibers composed of monosaccharide polymers, which undergo microbial fermentation in the intestines. This process produces molecules like SCFAs and peptidoglycan, which impact the innate immune system. It is suggested that prebiotics can reduce intestinal inflammation, endotoxaemia, and hypercytokinaemia, which might benefit critical illness. Additionally, they positively influence mucosal immunological homeostasis and barrier integrity [128, 129]. Probiotics, defined as live microorganisms that, when administered in adequate quantities, confer health benefits to the host, encompass various strains such as bifidobacteria, lactic acid bacteria, enterococci, and yeast [130, 131]. Their contributions include protecting the intestinal barrier, inhibiting pathogen proliferation, reducing bacterial translocation, improving lipid profiles, reducing uraemic toxins, modulating levels of serum pro-inflammatory cytokines, enhancing levels of serum anti-inflammatory cytokines, and promoting host immunomodulation to prevent infections [132–137].

Fecal microbiota transplantation, where fecal material from a healthy donor is transferred to a patient with disrupted gut microbiota, has shown therapeutic efficacy during critical illness by promoting the recovery of intestinal diversity, suppressing the growth of pathogenic bacterial communities in the gut, fostering competitive exclusion of pathogenic bacteria by the local intestinal microbiota, and restoring the host immune response. [138–142].

Indeed, shifting the focus of future research from conventional symptomatic treatments like lung protective MV is essential. Efforts should be redirected towards implementing more precise interventions to restore immune balance, mitigate inflammatory responses, and reestablish the ecological balance of the gut-lung axis while also preventing alterations of the lung and gut microbiome. Moreover, by adjusting the gut microbiota and fortifying the integrity of the intestinal barrier, crucial mechanisms can be activated to decrease bacterial migration, diminish exposure to endotoxins, and maintain overall immune stability, thereby having the potential to reduce both the frequency and severity of ARDS and the associated intestinal dysfunction.

## Conclusions

The strong correlation between the severity of nonpulmonary organ failure and ARDS underscores the importance of considering extrapulmonary manifestations in ARDS management and prognosis. With increasing ARDS severity, organ dysfunction intensifies, involving multiple systems. The gut-lung axis is acknowledged as a crucial component in the pathophysiology of ARDS, highlighting the bidirectional communication that entails bacterial and immune interactions between the gut and lungs within each respective compartment. Every kingdom and compartment plays a pivotal role in this interplay, thus influencing the prognosis of LI/ARDS. Among lung protective ventilation and maintenance of systemic haemodynamics, potential therapies aiming at restoring intestinal integrity, microbiome, and homeostasis balance between the two systems could significantly contribute to LI/ARDS management. While the gut-lung axis is acknowledged as crucial in ARDS pathophysiology, additional research is needed to clarify the bidirectional pathways of gut-lung interactions and their role in lung and extra-pulmonary injury. This understanding is essential for developing therapies that target maintaining intestinal integrity, microbiome, and homeostasis to manage LI/ARDS effectively.

## Data Availability

Not applicable.
